# High-fat diet feeding alters metabolic response to fasting/non fasting conditions. Effect on caveolin expression and insulin signalling

**DOI:** 10.1186/1476-511X-10-55

**Published:** 2011-04-13

**Authors:** Ana Gómez-Ruiz, Fermín I Milagro, Javier Campión, J Alfredo Martínez, Carlos de Miguel

**Affiliations:** 1 Department of Biochemistry and Molecular Biology, University of Navarra, Pamplona, Spain; 2 Department of Nutrition and Food Sciences, Physiology and Toxicology, University of Navarra, Pamplona, Spain

## Abstract

**Background:**

The effect of food intake on caveolin expression in relation to insulin signalling was studied in skeletal muscle and adipocytes from retroperitoneal (RP) and subcutaneous (SC) adipose tissue, comparing fasted (F) to not fasted (NF) rats that had been fed a control or high-fat (HF) diet for 72 days.

**Methods:**

Serum glucose was analysed enzymatically and insulin and leptin by ELISA. Caveolins and insulin signalling intermediaries (IR, IRS-1 and 2 and GLUT4) were determined by RT-PCR and western blotting. Caveolin and IR phosphorylation was measured by immunoprecipitation. Data were analysed with Mann-Whitney U test.

**Results:**

High-fat fed animals showed metabolic alterations and developed obesity and insulin resistance. In skeletal muscle, food intake (NF) induced activation of IR and increased expression of IRS-2 in control animals with normal metabolic response. HF animals became overweight, hyperglycaemic, hyperinsulinemic, hyperleptinemic and showed insulin resistance. In skeletal muscle of these animals, food intake (NF) also induced IRS-2 expression together with IR, although this was not active. Caveolin 3 expression in this tissue was increased by food intake (NF) in animals fed either diet. In RP adipocytes of control animals, food intake (NF) decreased IR and IRS-2 expression but increased that of GLUT4. A similar but less intense response was found in SC adipocytes. Food intake (NF) did not change caveolin expression in RP adipocytes with either diet, but in SC adipocytes of HF animals a reduction was observed. Food intake (NF) decreased caveolin-1 phosphorylation in RP but increased it in SC adipocytes of control animals, whereas it increased caveolin-2 phosphorylation in both types of adipocytes independently of the diet.

**Conclusions:**

Animals fed a control-diet show a normal response to food intake (NF), with activation of the insulin signalling pathway but without appreciable changes in caveolin expression, except a small increase of caveolin-3 in muscle. Animals fed a high-fat diet develop metabolic changes that result in insulin signalling impairment. In these animals, caveolin expression in muscle and adipocytes seems to be regulated independently of insulin signalling.

## Background

Obesity is a complex multifactorial condition that results from a combination of environmental (such as imbalanced eating habits and sedentary lifestyle) and neuroendocrine factors, coupled to a genetic predisposition [1]. Different genes have been related to obesity development, such as the three major isoforms of caveolin, Cav-1, Cav-2 and Cav-3 (18-24 kDa) [2]. Cav-1 is most abundantly expressed in terminally differentiated cells such as fibroblasts, epithelial and endothelial cells and adipocytes, where it is responsible for caveolae formation [3]. Cav-2 is coexpressed with Cav-1, while Cav-3 is the specific isoform of muscle tissue, although it has also been found in astrocytes and chondrocytes [4,5]. These proteins are the main structural components of caveolae and interact with signalling molecules through a characteristic scaffolding domain [6]. Enhanced cellular signalling within caveolae is facilitated due to the target-rich environment formed by the clustering of receptors and signalling molecules in the proximity of these membrane structures, thereby permitting a better controlled and more efficient signal transduction [7]. Insulin receptor (IR) is among those that can be located in caveolae and in fact, several studies have shown that, in adipocytes, Cav-1 is an important regulatory element stimulating IR signalling and linking insulin action to glucose uptake [8].

In obesity-related disorders, such as insulin resistance and type 2 diabetes, insulin signalling becomes altered, while adipose tissue develops chronic inflammation and hypoxia, conditions that affect gene expression through the associated oxidative stress and reactive oxygen species (ROS) production [9]. In regard to this, caveolin expression is highly dependent on proinflammatory factors such as TNF-alpha [10], and oxidative stress induces cellular senescence through activation of the Cav-1 promoter and upregulation of Cav-1 protein expression [11].

In addition, the two major targets of insulin action are skeletal muscle and adipose tissue [12]. White adipose tissue (WAT) serves as the main site for energy storage in the form of triglycerides, but also contributes to systemic glucose and lipid regulation acting as an endocrine organ [13]. The principal site of glucose uptake under insulin-stimulated conditions is skeletal muscle, being considered a primary site for insulin resistance [14]. An impairment of the initial steps in insulin signalling transduction pathways could contribute to the deficiency in insulin-stimulated glucose uptake in skeletal muscle, thus resulting in insulin resistance. In fact, different mechanisms have been described in relation to lipid-induced muscle insulin resistance, including acute free fatty acid elevation and prolonged lipid accumulation in muscle [15].

In previous studies, our group has demonstrated that caveolins are time-dependently regulated by diet-induced obesity [16]. In a late phase, after a prolonged time feeding on a high-fat diet, insulin resistance becomes apparent, accompanied by an impairment of Cav-3 and Cav-1 in skeletal muscle [17], whereas in adipocytes, Cav-1 and Cav-2 expression are increased, together with an inhibition of insulin signalling intermediaries [18].

The purpose of the present study is to deepen into the understanding of caveolin regulation in conditions of diet-induced obesity and insulin resistance, and their relation to insulin signalling in skeletal muscle and adipocytes. Given that fasting, the usual condition in animal studies before isolating samples, downregulates insulin secretion and signalling and therefore would influence caveolin physiology, we have compared for the first time caveolin regulation between fasted (F) and fed (non-fasted, NF) animals. We have analysed the response of skeletal muscle and adipocytes isolated from visceral and subcutaneous locations of lean (C, control) and high-fat (HF) diet-induced obese, insulin-resistant rats.

## Methods

### Animals, diets and experimental design

Male Wistar rats (250-300 g) were supplied by the Applied Pharmacobiology Center (University of Navarra, Spain) and housed under controlled conditions of temperature (22 ± 2°C), relative humidity (55 ± 10%) and 12 hours light cycle (8 a.m. to 8 p.m.). Sixteen animals were assigned to two different dietary groups for 72 days. The control group (C, n = 8) was fed standard laboratory pelleted diet (Harlam Iberica, Barcelona, Spain), providing 350 kcal/100 g, with about 10% of the energy as fat, 73% as carbohydrates and 17% as protein. A second group (n = 8) was fed a fat-rich cafeteria diet (HF), providing 430 kcal/100 g, with about 59% of the energy as fat, 32% as carbohydrates and 9% as protein, which was prepared with pate, chips, chocolate, bacon, biscuits and chow in a proportion of 2:1:1:1:1:1, as previously reported [19]. Animals had *ad libitum *access to food and water during the treatment. Weight gain and food/water intake were measured three times per week. Twelve hours before sacrifice 4 animals of each group were fasted (F) by food withdrawal (C-F and HF-F), whereas the other 4 were not fasted (NF), having free access to food (C-NF and HF-NF). After sacrifice by decapitation, truncal blood was collected and the gastrocnemius muscle and the retroperitoneal (RP) and subcutaneous (SC) white adipose fat pads were carefully excised and weighed. Muscle samples were immediately frozen in liquid nitrogen and stored at -80°C until use. RP and SC adipose samples were immediately processed for adipocyte isolation. All the procedures were performed according to national guidelines and under care of the Animal Care and Use Committee at the University of Navarra.

### Adipocyte isolation

RP and SC white fat samples were minced using fine scissors during 1 minute for RP and 2 minutes for SC [20]. Grinded tissue was digested at 37°C in KRBA buffer (Krebs-Ringer bicarbonate) containing collagenase type II (1.25 g/ml), during 30 minutes for RP and 40-60 minutes for SC, the digestion was stopped adding 24 ml of KRBA buffer. Adipocytes were separated from stromal cells by passing through mesh tissue, and washed three times at 37°C with 15 ml of KRBA buffer during 5-10 minutes before freezing in liquid nitrogen to keep them stored al -80°C until use.

### Analysis of blood samples

Serum glucose was analyzed using an Autoanalyzer (COBAS Roche Diagnostic, Basel, Switzerland) by enzymatic routine procedures. Serum insulin (Mercodia A-B, Uppsala, Sweden) and leptin (Rat Leptin ELISA Kit, Linco Research, St Charles MO, USA) levels were measured by ELISA. HOMA index (Homeostasis Model Assessment) was calculated as fasting insulin (μU/ml) × fasting glucose (mmol/L)/22.5.

### Reverse Transcripcion (RT)-PCR

Total RNA was extracted from frozen skeletal muscle and RP and SC adipocytes, with Trizol (Invitrogen, Carlsbad CA, USA). Contaminating genomic DNA was removed by treatment with DNase (DNA-free™ kit, Applied Biosystems, Austin TX, USA) and purified total RNA was used as a template to generate first strand cDNA synthesis using M-MLV reverse transcriptase (Invitrogen, Carlsbad CA, USA) and random hexamers (Applied Biosystems, Austin TX, USA) as primers. Quantitative real-time PCR was performed as described by the provider (Applied Biosystems, Austin TX, USA) using an ABI-PRISM 7300 HT Sequence Detection System and Taqman probes for rat Cav-1, Cav-2, Cav-3, GLUT4, IR, IRS-1 and IRS-2. GAPDH was used as internal control for RT-PCR efficiency and subsequent normalization. The results were calculated by the 2^-ΔΔCt ^method [21].

### Western Blot Analyses

Skeletal muscle samples were homogenized with HES lysis buffer containing 20 mM HEPES, 5 mM EDTA, 250 mM sucrose, and 1X protease inhibitor mix (Sigma-Aldrich, Madrid, Spain) pH 7.5. RP and SC adipocytes samples were homogenized with the same buffer by pippeting. Cell lysates were cleared by centrifugation (12,000 g) at 4°C for 45 minutes and supernatants were stored at -80°C. Protein content was determined with bincinchoninic Acid Kit (Sigma-Aldrich Madrid, Spain). For skeletal muscle, 10 to 15 μg, and for adipocytes, 20 to 50 μg protein were separated by SDS-PAGE gel electrophoresis at 130-150 V for 45 minutes, and electro-transferred to nitrocellulose membranes (Schleicher & Schuell, Amersham Biosciences, Piscataway NJ, USA) at 300 mA for one hour. Non-specific binding sites were blocked for 2 h at room temperature with Tris-base buffer containing 0.1% Tween 20 and 4% non-fat milk except for Cav-2 membranes that were blocked with Tris-base buffer containing only 10% Tween 20. After blocking, membranes were incubated overnight with specific primary antibodies at suitable dilutions. Secondary HRP-conjugated antibodies were added for one hour and the signal developed with the Super Signal West Pico Chemiluminescence Substrate (Pierce Biotechnology, Inc., Rockford IL, USA). Membranes were exposed to Hyperfilm ECL blotting (Amersham Biosciencies, Piscataway NJ, USA). When necessary, blots were stripped by immersion in Red-Blot Plus solution 10X (Millipore, Millipore, Billerica MA, USA) and reprobed with different antibodies, using the same procedure described above. Protein bands were quantified by Quantity One data analyzer software 4.6.3 (Bio-Rad, Muenchen, Germany) and normalized to the β-actin signal as internal control. Cav-1 (1/50,000), Cav-2 (1/20,000) for skeletal muscle and (1/250) for RP and SC adipocytes, and Cav-3 (1/10,000) antibodies were supplied by Santa Cruz Biotechnology Inc., (Santa Cruz CA, USA). Insulin receptor IR (1/10,000) and Phosphotyrosine (1/10,000) for skeletal muscle and (1/5,000) for RP and SC adipocytes, antibodies were obtained from Cell Signalling Technology, (Danvers MA, USA) and β-actin (1/10,000) antibody was purchased from Sigma-Aldrich (St. Louis MO, USA). The secondary antibodies were anti-mouse (1/10,000) from Amersham Biosciences-GE Healthcare (Piscataway, NJ, USA), anti-rabbit (1/10,000), from Sigma-Aldrich (St. Louis MO, USA) and anti-goat (1/10,000), from Santa Cruz Biotechnology Inc. (Santa Cruz CA, USA).

### Inmunoprecipitation

Samples containing 250-500 μg of total protein were incubated with antibodies against Cav-1 (1/100 v/w), Cav-2 (1/100 v/w), Cav-3 (1/100 v/w) and IR (1/50 v/w) overnight at 4°C on a rotating device, followed by addition of Protein A-G PLUS Agarose (Santa Cruz Biotechnology Inc., Santa Cruz CA, USA) for 2 hours in the same conditions. Immunoprecipitation complexes were washed three times with 500 μl of HES lysis buffer containing 20 mM HEPES, 5 mM EDTA, 250 mM sucrose and 1X protease inhibitor (Sigma-Aldrich Madrid, Spain) pH = 7.5 for skeletal muscle samples, and with 500 μl of lysis buffer (0.25% sodium deoxicholate, 50 mM Tris-HCl pH 7.5, 150 mM NaCl, 1% Triton X-100, 5 mM EDTA, 2 mM NaF, 2 mM sodium ortovanadate, 6 mM octil-glucoxide and 1X protease inhibitor mix (Sigma-Aldrich, Madrid Spain) for adipocyte samples. Supernatants were discarded and pellets resuspended in 20 μl Laemmli sample buffer 1X. After 7 min boiling, samples were centrifuged and analyzed by Western blotting with the corresponding antibodies as described above. Phosphorylated IR and caveolin bands were normalized with total IR or caveolin as reference.

### Data analysis

The results were expressed as the mean ± S.E.M. Data were compared using Mann-Whitney *U *test. All analyses were performed using SPSS version 15.0 for Windows.

## Results

### Body, tissue weight and blood determinations

As previously reported using the same dietary model [17], body and adipose depot weights of the animals fed on the HF-diet were significantly higher than those fed on the control diet. However, no differences were found between the fasted (F) and non-fasted (NF) groups of animals (Table 1). Only gastrocnemius muscle weight decreased by fasting independently of the diet (Table 1), probably due to glycogen depletion and protein mobilization from muscle tissue [22].

**Table 1:** Body and tissue weights and metabolic determinations in the four experimental groups

	C		HF	
	**F**	**NF**	**F**	**NF**

**Body Weight (g)**	424 ± 7	431 ± 23	508 ± 33^#^	538 ± 18 ^t^

**Skeletal Muscle (g)**	2.01 ± 0.33	2.51 ± 0.06*	1.93 ± 0.04	2.64 ± 0.10*

**Retroperitoneal fat (g)**	7.99 ± 1.30	9.90 ± 1.70	20.49 ± 2.75^#^	17.64 ± 2.57 ^t^

**Subcutaneous fat (g)**	8.15 ± 1.54	10.99 ± 2.12	17.41 ± 2.31^#^	20.68 ± 6.06

**Glucose (mg/dL)**	104.45 ± 4.33	124 ± 6.22*	125.02 ± 5.97 ^t^	123.3 ± 3.47

**Insulin (ng/mL)**	0.45 ± 0.09	2.05 ± 0.14*	1.80 ± 0.47^#^	2.50 ± 0.47 ^T^

**Leptin (ng/mL)**	3.53 ± 0.84	18.94 ± 4.06*	17.87 ± 3.54^#^	20.93 ± 5.78

C: control diet fed animals. HF: high-fat diet fed animals. F: Fasted animals. NF: Non fasted animals. Data are given as means ± SEM. Groups were compared using the Mann-Whitney U test: (F vs NF), T < 0.10; *p < 0.05; (C-F vs HF-F and C-NF vs HF-NF), t < 0.10, # p < 0.05.

Animals grown on the control diet responded normally to feeding (NF) and showed increased levels of glucose, insulin and leptin as a consequence of food intake (Table 1). On the other hand, animals fed on the HF-diet were metabolically altered showing insulin resistance, hyperglycemia, hyperinsulinemia and hyperleptinemia. Therefore, the circulating levels of glucose, insulin and leptin were either not affected (glucose) or only slightly increased (insulin and leptin) by continuous feeding (NF) (Table 1). These results suggests a preliminary state of insulin resistance in these animals, as indicated by their significantly higher (p < 0.01) HOMA index: (C) 2.99 ± 0.68, (HF) 14.07 ± 3.99.

### mRNA expression of insulin pathway intermediaries

**Figure 1:**
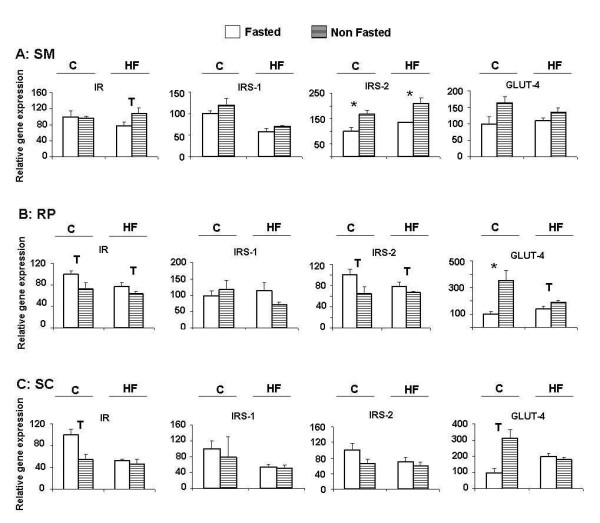
**mRNA levels of IR, IRS-1, IRS-2 and GLUT4**. C: control diet fed animals. HF: high-fat diet fed animals. F: Fasted animals. NF: Non fasted animals. (A) Skeletal muscle (SM). (B) Retroperitoneal adipocytes (RP). (C) Subcutaneous adipocytes (SC). Data are means ± SEM of the ratio between each gene and GAPDH expression and are referred to gene expression in control-diet fed, fasted group (C-F = 100). Groups (F vs NF) were compared using the Mann-Whitney U test: T < 0.10; *p < 0.05.

**Figure 2:**
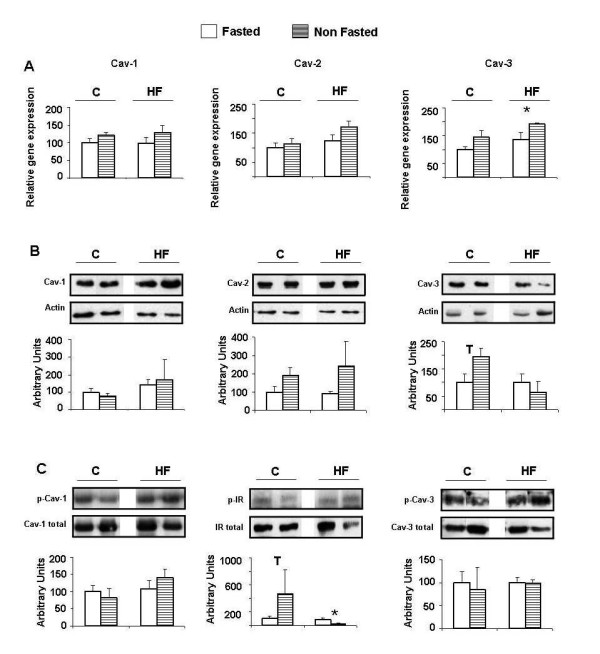
**Caveolin expression and caveolin and insulin receptor (IR) phosphorylation in skeletal muscle**. C: control diet fed animals. HF: high-fat diet fed animals. F: Fasted animals. NF: Non fasted animals. (A) mRNA levels. Data are means ± SEM of the ratio between each gene and GAPDH expression and are referred to gene expression in control-diet fed, fasted group (C-F = 100). (B) Protein levels. Data are means ± SEM of the ratio between each caveolin and actin and are referred to expression in control-diet fed, fasted group (C-F = 100). (C) Phosphorylation levels. Data are means ± SEM of the ratio between each phosphoprotein and total caveolin or IR and are referred to phosphorylation level in control-diet fed, fasted group (C-F = 100). Groups (F vs NF) were compared using the Mann-Whitney U test: T < 0.10; *p < 0.05.

In skeletal muscle, control-fed animals showed an increase in IRS-2 expression when not fasted (NF), which was maintained in HF-fed animals (Figure 1A). Although not significant, GLUT4 expression was also higher in NF control animals, but this difference was lower in HF-fed animals (Figure 1A). GLUT4 mRNA levels were also higher in adipocytes of NF control animals, whether they came from the RP or the SC pads, and this difference was also reduced in HF-fed animals, specially in SC adipocytes (Figure 1B, C). On the contrary, in RP adipocytes, IR and IRS-2 expression were slightly down-regulated in NF animals independently of the diet (Figure 1B). In SC adipocytes a similar trend was observed, but this difference only reached marginal significance for IR in control-fed animals (Figure 1C). As expected, muscle IR-phosphorylation was higher in the control-diet fed NF animals as compared to the fasted ones. However, this activation decreased in the NF animals fed the HF diet (Figure 2C).

### Caveolin expression and activation in skeletal muscle

Neither expression of Cav-1 and Cav-2 nor phosphorylation level of Cav-1 were affected by the feeding status with either diet (Figure 2). Only Cav-3 expression seemed to be higher in NF animals, showing increased mRNA levels in the HF-fed group and higher protein levels in the control-fed group (Figure 2).

### Caveolin expression and activation in adipocytes from retroperitoneal white adipose tissue

**Figure 3:**
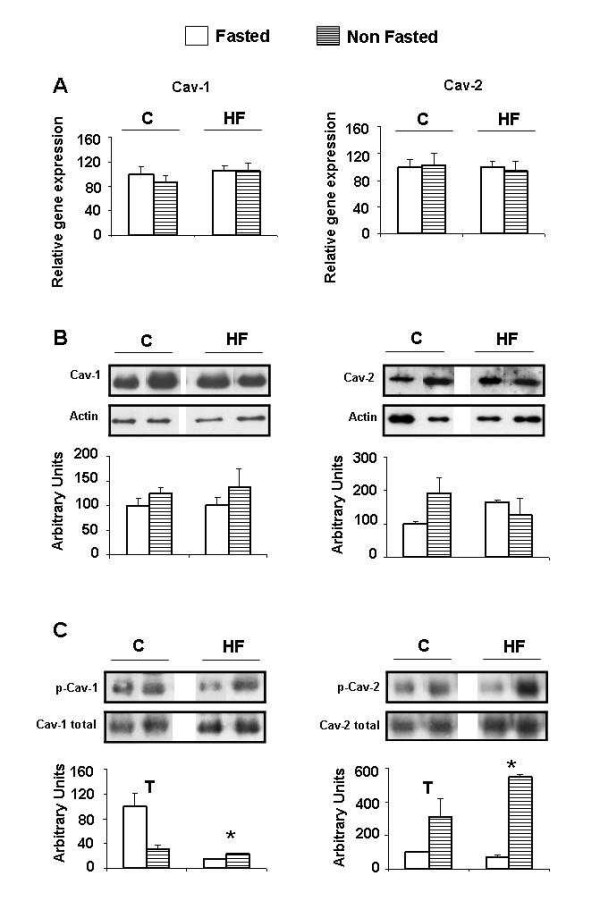
**Caveolin expression and phosphorylation in retroperitoneal adipocytes**. C: control diet fed animals. HF: high-fat diet fed animals. F: Fasted animals. NF: Non fasted animals. (A) mRNA levels. Data are means ± SEM of the ratio between each gene and GAPDH expression and are referred to gene expression in control-diet fed, fasted group (C-F = 100). (B) Protein levels. Data are means ± SEM of the ratio between each caveolin and actin and are referred to expression in control-diet fed, fasted group (C-F = 100). (C) Phosphorylation levels. Data are means ± SEM of the ratio between each phosphoprotein and total caveolin and are referred to phosphorylation level in control-diet fed, fasted group (C-F = 100). Groups (F vs NF) were compared using the Mann-Whitney U test: *p < 0.05.

Caveolin expression did not change in NF animals either at mRNA or protein level (Figure 3A, B). However, significant variations in caveolin activation were observed when their state of phosphorylation was measured. Cav-1 phosphorylation was reduced in NF animals fed the control diet but increased in animals fed the HF diet (Figure 3C). On the other hand, Cav-2 showed enhanced phosphorylation in NF animals independently of the diet (Figure 3C).

### Caveolin expression and activation in adipocytes from subcutaneous white adipose tissue

**Figure 4:**
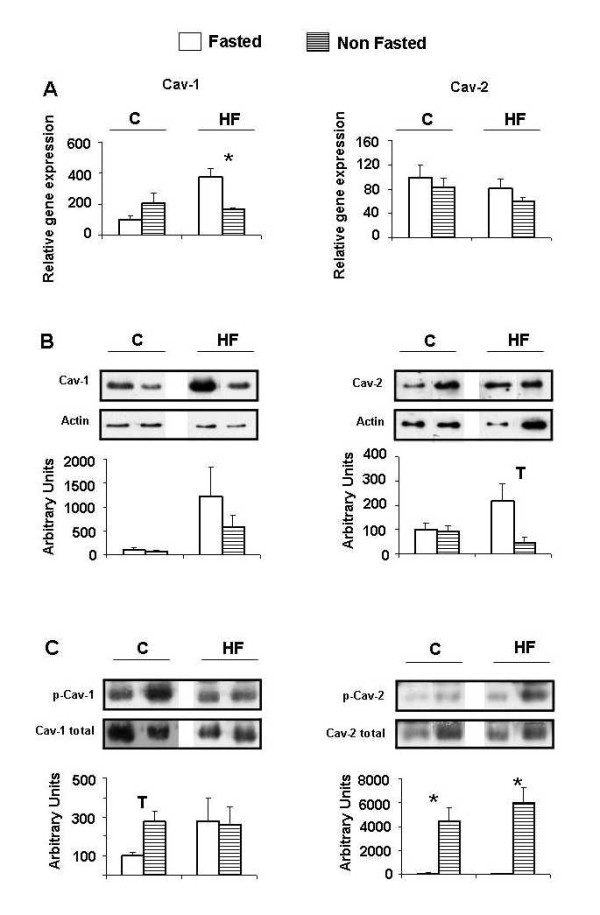
**Caveolin expression and phosphorylation in subcutaneous adipocytes**. C: control diet fed animals. HF: high-fat diet fed animals. F: Fasted animals. NF: Non fasted animals. (A) mRNA levels. Data are means ± SEM of the ratio between each gene and GAPDH expression and are referred to gene expression in control-diet fed, fasted group (C-F = 100). (B) Protein levels. Data are means ± SEM of the ratio between each caveolin and actin and are referred to expression in control-diet fed, fasted group (C-F = 100). (C) Phosphorylation levels. Data are means ± SEM of the ratio between each phosphoprotein and total caveolin and are referred to phosphorylation level in control-diet fed, fasted group (C-F = 100). Groups (F vs NF) were compared using the Mann-Whitney U test: *p < 0.05.

In contrast to adipocytes from the RP pad, some changes were observed in caveolin expression in SC adipocytes. Thus, NF animals fed the HF diet showed decreased levels of Cav-1 mRNA and of Cav-2 protein. A reduction of Cav-1 protein and Cav-2 mRNA levels were also observed in the same groups of animals, but did not reached statistical significance (Figure 4A, B). Interestingly, Cav-2 phosphorylation was increased in NF animals independently of the diet (Figure 4C), following the same pattern as observed in RP adipocytes. On the other hand, Cav-1, whose phosphorylation increased only in NF control-fed animals (Figure 4C), seemed to follow the opposite pattern to that observed in RP adipocytes.

## Discussion

In agreement with previous studies [17,18], the current results indicate that the animals fed on a high-fat cafeteria diet for an extended period of time, besides becoming overweight, show hyperleptinemia, hyperglycemia, hyperinsulinemia and develop insulin resistance. This outcome is an indication of an altered metabolic state related to obesity development. These determinations are usually made in animals fasted overnight before being sacrificed (F), so that the differences in food intake between them during the previous hours will not influence the levels found. In the present study, a group of animals had free access to food until the moment of sacrifice and therefore were not fasted overnight (NF). These NF animals, fed a control-diet, showed the expected higher glucose and insulin levels as a consequence of food intake, which constitutes the normal metabolic response. These animals also showed increased leptin levels, explained as a satiety signal [23] (Table 1).

On the other hand, the NF animals that were fed the HF-diet experienced the mentioned insulin resistant metabolic state, which is characterized by higher levels of glucose, insulin and leptin. In this case, the continuous food availability produced smaller, although still apparent, increases in insulin and leptin levels, but no changes in circulating glucose (Table 1).

### Effect of non fasting in skeletal muscle

In normal conditions, skeletal muscle is responsible for 80% of blood glucose clearance, whereas after several hours of fasting the brain becomes the main destination for circulating glucose [24]. NF control-diet fed animals had high serum glucose levels and an active insulin signalling machinery that promotes glucose entrance in muscle cells. In fact, in these animals increased IR-phosphorylation and IRS-2 expression were found (Figures 1A and 2C). GLUT4 expression was also slightly higher, although the difference did not reach statistical significance (Figure 1). Although IRS-1 in muscle and adipose cells is known as the main IR substrate that mediates glucose uptake through PKB/Akt activation and GLUT4 translocation to plasma membrane [25], in liver, it has been described that IRS-1 and IRS-2 may complement each other in insulin signalling [26]. Therefore, the observed increase in IRS-2 expression (Figure 1) could reflect an attempt to improve insulin signalling in muscle cells. This response could be stressed in HF-diet fed animals under altered metabolic conditions, as suggested by the increased IR expression in NF individuals. Nevertheless in these animals this regulatory mechanism seems to be ineffective, since GLUT4 expression was not modified (Figure 1A) and blood glucose remained high (Table 1). As a matter of fact IR phosphorylation was diminished in NF HF-diet fed animals (Figure 2C).

Since Cav-3 is the main isoform expressed in muscle tissue, being functionally equivalent to Cav-1, it is not surprising that only Cav-3 expression seems to be affected in NF animals. Increased Cav-3 expression in normal conditions, as observed in NF control-diet fed animals, may be related to improved insulin signalling [17], although Cav-3 phosphorylation does not change (Figure 2C). In NF HF-diet fed animals Cav-3 mRNA level was higher, but this increase in expression would also be ineffective in glucose metabolism as explained before. Therefore, in agreement with previous results [17], it seems that in HF-diet fed animals, regulation of Cav-3 expression is not related to an impaired insulin signalling cascade. In regard to this, it has been reported that Cav-3 expression may be induced by oxidative stress through p38MAPK [27]. Indeed, previous studies have reported that animals fed on a high fat diet show increased oxidative stress in muscle tissue [28].

### Effect of non fasting in adipocytes of white adipose tissue

Adipose tissue is considered a multifunctional organ having a critical role in metabolism and energy balance regulation, and enlargement of adipose tissue depots is the most characteristic feature of obesity [29]. However, the distribution of body fat appears to be even more important than the total amount of fat. In this way, abdominal adiposity is much more closely associated with insulin resistance, type 2 diabetes, hypertension, and dyslipidemia than subcutaneous fat mass [30]. Visceral adipose tissue is also thought to be not only metabolically more active than subcutaneous, showing higher insulin-stimulated glucose uptake [31], but also more intensely affected by obesity-related inflammation and oxidative stress [32].

In the current work, we have studied caveolin expression in relation to insulin signalling in adipocytes isolated from the visceral (RP) and SC adipose depots in animals fed a HF-diet that were fasted (F) or not fasted (NF) before sacrifice.

In RP adipocytes it was observed that IR and IRS-2 expression were downregulated in NF control-diet fed animals whereas GLUT4 was upregulated. Analogous results were obtained in SC adipocytes, although in this case IRS-2 expression was not affected (Figure 1B, C), and a similar observation was made in brown adipose tissue [33]. Control-diet fed animals have functional insulin signalling transmission and the GLUT4 increase may be related to insulin stimulation after feeding. In addition, Desbuqois et al. [34] reported that hyperinsulinemia can stimulate IR degradation and a decrease in IR mRNA level, and the NF control-diet fed animals, as expected, do actually exhibit higher insulin level (Table 1).

Non-fasted animals fed the HF-diet showed very similar results in RP adipocytes, but the increase in GLUT4 expression was notably less marked (Figure 1B). Since these animals have become hyperinsulinemic, blood insulin increment caused by food intake did not substantially modify the situation (Table 1), and the drop in IR or IRS-2 expression was also less pronounced (Figure 1). These animals have an altered insulin response that would diminish glucose uptake despite a GLUT4 expression increase. Therefore, GLUT4 expression seems to be regulated independently of the insulin signalling cascade intermediaries. In relation to this, chronic local inflammation, cell hypoxia, and the associated production of ROS and oxidative stress described in adipose tissue of obese subject [9], have been described as factors that can upregulate GLUT4 in adipocytes [35]. On the other hand, in SC adipocytes, a lack of effect was observed on the insulin intermediaries gene expression in the NF animals fed on the HF-diet (Figure 1C). This outcome is probably due to the fact that SC fat is metabolically less active, and therefore less sensitive to nutritional variations, than RP fat [36-38].

In RP adipocytes expression of neither of the caveolins seemed to be influenced by food intake (NF) independently of the diet (Figure 3). However, in SC adipocytes a decrease of Cav-1 mRNA and of Cav-2 protein was observed in NF HF-diet fed animals (Figure 4). In regard to this, differences in caveolin regulation have been observed between visceral and SC adipocytes from obese humans [39].

In contrast with muscle, food intake (NF) provoked changes in the phosphorylation state of caveolins in adipose tissue. Cav-1 is the isoform more directly implicated in insulin signalling in adipose tissue, therefore, under normal conditions (control-diet), increased activation of Cav-1 when glucose level rises by nutrient intake (NF), would improve insulin signal. We have observed this behaviour in the less insulin sensitive SC adipocytes (Figure 4C). However, in RP adipocytes this response might not be necessary, and indeed Cav-1 became less phosphorylated by nutrient intake (Figure 3C). This effect could be related to the reduction in IR and IRS-2 expression associated to glucose level elevation produced by food intake as mentioned before. In fact, high glucose serum levels have been shown to downregulate Cav-1 expression in Schwann cells [40]. Cav-2 does not seem to be directly involved in insulin signalling and is considered a structural aid for functional caveolae formation. In this sense, increased Cav-2 phosphorylation by food intake in normal conditions, as observed in both RP and SC adipocytes (Figures 3C and 4C), could be considered a mechanism to improve insulin signalling.

In the altered metabolic conditions of HF-diet fed animals, caveolin phosphorylation would not be regulated in coordination with the impaired insulin signalling cascade, but it could conceivably be more related to the increased inflammation and oxidative stress conditions associated with obesity in adipose tissue. As a matter of fact, it has been described that Cav-1 phosphorylation can be mediated by p38MAPK and Scr through a pathway induced in response to oxidative stress [41]. In regard to this, oxidative stress response elements have been described in the promoter of caveolin-1 [42], which indeed responds to oxidative stress with an increase in its expression [43]. With respect to inflammation, LPS and different proinflamatory cytokines such as TNF-alpha and IL-1 have been shown to induce the upregulation of Cav-1 through the NF-Kappa B pathway [44]. The link between Cav-1 and these phenomena is confirmed by the observation that, the loss of Cav-1 in bone marrow-derived stromal cells from Cav-1 deficient mice, induces oxidative stress and mimics a pseudo-hypoxic state that leads to inflammation in the tumor stromal microenvironment [45]. In relation to our results it has been shown that transient hyperglycemia (i. e. nutrient intake) induces an increase in plasma IL-6, TNF-alpha, and IL-18 mediated by oxidative stress [46]. In the current work, we report that nutrient intake (NF), in a comparable situation, provokes increased phosphorylation of both caveolins in RP adipocytes and only of Cav-2 in SC cells (Figures 3C and 4C).

These differences also strengthen the idea that both types of adipose tissue are not metabolically equivalent and have different responses in accordance with their physiological role [47].

## Conclusions

Animals fed the control-diet show a normal metabolism, and food intake (NF) provokes elevation of serum glucose, insulin and leptin levels. This in turn would cause the activation of the insulin signalling pathway in muscle and adipose tissue, which will result in increased glucose uptake through the stimulation of GLUT4 activity in the cell membrane. Caveolin expression does not seem to be critically affected except in muscle, where a slight increase in Cav-3, the main isoform in this tissue, is observed.

On the other hand, animals fed the HF-diet develop a prediabetic altered metabolism in which insulin signalling is impaired. In this condition, caveolin expression in muscle and adipose tissue in response to food intake (NF) seems to be regulated independently of insulin signalling, as we have reported previously in fasted animals [18]. In summary our data clearly show that caveolins respond to nutritional changes and strengthen the role of these proteins in the regulation of energy metabolism.

## Competing interests

The authors (AG-R; FIM; JC; JAM; CdM) report no competing interests.

## Authors' contributions

AG-R carried out the care and maintenance of the animals and all the experimental determinations and drafted the manuscript. FIM helped with the care and maintenance of the animals and the RT-PCR determinations, participated in the experimental design, in the revision and interpretation of the data and reviewed the manuscript. JC helped with the care and maintenance of the animals and participated in the interpretation of the data and revision of the manuscript, JAM participated in the interpretation of the data and revision of the manuscript, CdM helped with the immunological determinations, participated in the experimental design, in the revision and interpretation of the data and drafted and reviewed the manuscript. All authors have read and approved the final manuscript.
